# Super-Resolution Ultrasound Localization Microscopy Using High-Frequency Ultrasound to Measure Ocular Perfusion Velocity in the Rat Eye

**DOI:** 10.3390/bioengineering10060689

**Published:** 2023-06-06

**Authors:** Hasan Ul Banna, Benjamin Mitchell, Stephen Chen, Joel Palko

**Affiliations:** 1 Ophthalmology and Visual Sciences, West Virginia University, Morgantown, WV 26505, USA; 2 School of Medicine, West Virginia University, Morgantown, WV 26505, USA

**Keywords:** glaucoma, intraocular pressure, ocular hemodynamics, ocular vasculature, super-resolution ultrasound, ultrasound localization microscopy

## Abstract

Imaging of the ocular vasculature can provide new insights into the pathophysiology of ocular diseases. This study proposes a novel high-frequency super-resolution ultrasound localization microscopy (SRULM) technique and evaluates its ability to measure in vivo perfusion changes in the rat eye at elevated intraocular pressure (IOP). A 38.4 MHz center frequency linear array transducer on a VisualSonics Vevo F2 imaging platform was used to collect high frame rate (1 kHz) radiofrequency data of the posterior rat eye following systemic microbubble contrast injection. Following clutter and spatiotemporal non-local means filtering, individual microbubbles were localized and tracked. The microbubble tracks were accumulated over 10,000 frames to generate vascular images quantifying perfusion velocity and direction. Experiments were performed using physiologic relevant controlled flow states for algorithm validation and subsequently performed in vivo on the rat eye at 10 mm Hg IOP increments from 10 to 60 mm Hg. The posterior vasculature of the rat eye, including the ophthalmic artery, long posterior ciliary arteries and their branches, central retinal artery and retinal arterioles and venules were successfully visualized, and velocities quantified at each IOP level. Significant reductions in arterial flow were measured as IOP was elevated. High-frequency SRULM can be used to visualize and quantify the perfusion velocity of the rat eye in both the retrobulbar and intraocular vasculature simultaneously. The ability to detect ocular perfusion changes throughout the depth of the eye may help elucidate the role ischemia has in the pathophysiology of ocular diseases such as glaucoma.

## 1. Introduction

Intraocular pressure (IOP) is the cardinal modifiable risk factor for the development and progression of glaucoma [1,2]. However, it remains unclear how IOP initiates and propagates the cascade of events leading to retinal ganglion cell death [3,4]. Converging evidence suggests that IOP-induced alterations to both the connective tissue and vasculature of the optic nerve head (ONH) are detrimental to the health of retinal ganglion cells [4,5,6,7]. Reduced perfusion to the ONH has been investigated as a contributor to glaucoma pathophysiology. Several epidemiologic studies have shown that reduced ocular perfusion pressure (OPP), generally defined as the mean arterial blood pressure to the eye minus IOP, is associated with glaucoma prevalence and progression [8,9,10,11,12]. Understanding the role blood flow and its autoregulation have in glaucoma pathophysiology has been aided by a variety of methodologies, with the goal of quantifying perfusion to the ONH.

Current clinically available optical vascular imaging modalities such as optical coherence tomography angiography (OCT-A) and laser speckle contrast imaging (LSCI) have provided insight into perfusion of the retina, peripapillary choroidal and prelaminar regions of the ONH. OCT-A has emerged as a potential clinical tool to estimate perfused vascular density of the microcirculation around the superficial ONH. Using OCT-A to evaluate the deep peripapillary choroidal vasculature, studies have established microvascular dropout and reduced vessel density as biomarkers for longitudinal glaucoma progression, and that lowering IOP can improve this microcirculation [13,14,15,16]. Using LSCI, relative dynamic (i.e., systolic and diastolic) blood flow velocities of the superficial ONH can be measured. Initial clinical cross-sectional studies using LSCI have shown statistically significant differences in blood flow velocities between glaucoma, glaucoma suspect and control eyes [17]. These studies have provided supportive evidence that perfusion to different regions of the ONH may have an important pathologic role in some eyes with glaucoma. However, currently available optical techniques for quantifying ONH perfusion are unable to evaluate blood flow within the retrobulbar and posterior ciliary arteries that anastomose to the laminar region of the ONH, where glaucomatous damage is thought to first occur [18,19]. There is a need for non-invasive ways to measure blood flow to the retina, choroid, ONH and retrobulbar vessels to enable a better understanding of glaucoma pathophysiology in both patients and experimental research. The use of ultrasound has been applied to image the vasculature of the eye to overcome the penetration limitations of optical methods; however, the resolution of ultrasound techniques remains a major barrier for quantifying blood flow outside of larger arteries and veins.

Clinical studies using ultrasound color doppler imaging (CDI) to measure retrobulbar blood flow in relationship to glaucoma found increased resistive indices of the short posterior ciliary arteries to be associated with progression in eyes with primary open angle glaucoma (POAG) [20]. Other cross-sectional clinical studies found reduced flow velocities and higher resistive indices in normal tension glaucoma and POAG eyes compared to controls [21,22,23]. Silverman et al. [24] showed reductions in the arterial velocity and vascular density, with increases in resistive and pulsatility indices using plane wave doppler imaging in rat eyes with acute IOP elevations. Doppler ultrasound imaging of the posterior eye, however, is limited in its resolution and sensitivity to discriminate perfusion changes in small blood vessels.

To overcome the diffraction limit of ultrasound, super-resolution ultrasound localization microscopy (SRULM) has been used to image and quantify perfusion in the microvasculature of various organ systems. Similar in concept to super-resolution optical techniques such as stochastic optical reconstruction microscopy and fluorescence photoactivated localization microscopy, SRULM produces sub-diffraction images by localizing and accumulating the center positions of gas-filled microbubbles (MB) standardly used as ultrasound contrast agents [25]. These bubbles can be tracked frame to frame with a variety of tracking methods to quantify perfusion velocity and direction to improve the resolution of the ultrasound system during vascular imaging while maintaining the penetration depth of ultrasound. The increase in frame rate provided by ultrafast ultrasound imaging, using techniques such as plane-wave emissions, provides the ability to track MBs in both high and low velocity flow states.

Successful applications of SRULM have been demonstrated clinically and in animal models. Huang et al. [26] demonstrated the feasibility of SRULM to image vasculature down to a resolution of 57.5 µm using a 3.6 MHz transducer in several human organs in vivo. Opacic et al. [27] has shown the ability of SRULM to characterize the vasculature of breast tumors in cancer patients. A broader use of SRULM has been applied in animal models of various diseases. Vascular and perfusion metrics with the ability to differentiate tumor types and treatment response has been derived from SRULM imaging in rodent cancer models [28,29]. Chen et al. [30] and Andersen et al. [31] have investigated changes in kidney blood flow related to ischemia-reperfusion damage in mouse and rat kidneys, respectively.

Qian et al. [32] has utilized SRULM to reveal reductions in vascular density and perfusion velocity in a posterior rabbit eye during acute IOP elevations. Morisset et al. [33] showed the use of SRULM and functional ultrasound in the rat eye to measure the neurovascular response of the retina. These preliminary animal studies using SRULM show its potential to provide the penetration depth required to image the vasculature of the eye thought to be most relevant to glaucoma while maintaining resolutions comparable to optical methods. SRULM also provides absolute velocity metrics compared to the relative velocities produced by techniques such as LSCI, and it is theoretically less dependent on the imaging angle compared to doppler ultrasound. In this report, we describe the use of SRULM using a high-frequency (38.4 MHz) ultrasound transducer to image the vasculature of the posterior segment and retrobulbar regions of in vivo rat eyes.

## 2. Materials and Methods

### 2.1. Ultrasound Instrumentation

Beamformed ultrasound radiofrequency data sampled at 96 MHz were acquired using a 38.4 MHz (25–57 MHz bandwidth) center frequency linear array ultrasound probe (UHF57x) operated by the Vevo F2 ultrasound imaging system (FUJIFILM VisualSonics, Toronto, ON, Canada). The power was determined empirically for our imaging depth by adjusting until MB density decreased secondary to ultrasound-induced disruption. The focused ultrasound system was used to collect data at a frame rate of 1 kHz with an image width of 5 mm. A total of 10,000 continuous frames were collected for each super-resolved image.

### 2.2. Controlled Flow Experiments

We characterized the performance of our ULM algorithms by first using a silicone flow phantom with an inner diameter of 310 µm and outer diameter of 640 µm (Freudenberg Medical, Carpinteria, CA, USA). The tubing was connected to a syringe pump (PHD Ultra, Harvard Apparatus, Holliston, MA, USA) to control perfusion velocity. The perfusate consisted of USphere Prime ultrasound contrast MBs (Trust Bio-sonics, Taiwan) at a 1/50 times dilution to the original concentration (2.5 × $10^{10}$ MBs/mL) in 0.9% saline solution. The typical diameter of the USphere perfluoropropane MB is 1.1–1.4 µm. Discrete velocities of 10 mm/s, 15 mm/s, 20 mm/s, 40 mm/s, 60 mm/s, 90 mm/s, 120 mm/s and 150 mm/s were used to evaluate the algorithm along a velocity spectrum containing low-flow and high-flow states. The transducer was oriented parallel to the flow through the tubing during RF data acquisition.

### 2.3. In Vivo Rat Eye Imaging

**Figure 1:**
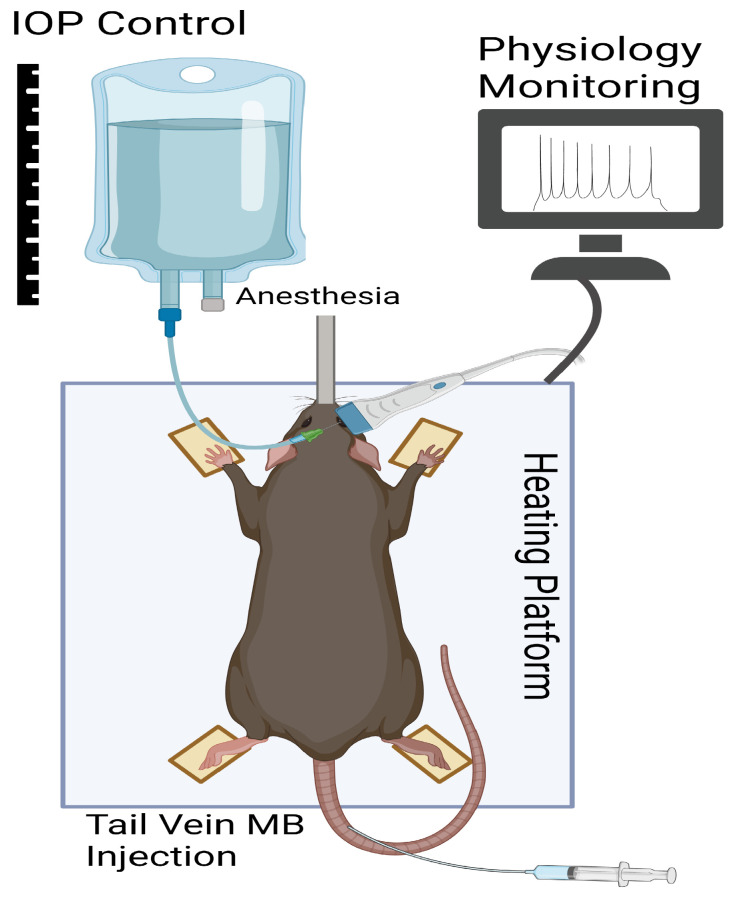
Schematic of experimental setup. Intraocular pressure (IOP) was controlled via cannulation of the anterior chamber of the rat eye with a 32-gauge needle through the superior peripheral cornea. Vitals, including heart rate, electrocardiogram and respiratory rate, were monitored via the built-in physiologic monitoring of the Vevo F2 system. The ultrasound probe was oriented horizontally. Figure created with BioRender.com (accessed on 12 March 2023).

The right eye of retired female breeder Brown Norway rats (n = 7), aged 4 to 6 months, were imaged in vivo with adherence to the guideline by the West Virginia University Institutional Animal Care and Use Committee (IACUC) and the ARVO Statement for the Use of Animals in Ophthalmic and Vision Research. The rats were anesthetized by induction with 5% isoflurane, which was then reduced to 2.5% during imaging, using 100% $O_{2}$ as the carrier gas. Animals were placed on a heated imaging table with a manual x-y-z linear stage and vitals were continuously monitored. Local anesthetic consisting of 0.5% proparacaine HCl (Alcon Inc., Ft. Worth, TX, USA) was placed in both eyes of the animal followed by the application of GenTeal (0.3% hypromellose, Alcon Inc., Geneva, Switzerland) for lubrication. Lateral tail vein access was obtained using a 25 gauge needle with extension tubing (Instech Laboratories, Philadelphia, PA, USA). In all experiments, a 0.05 mL bolus of 1:10 normal saline diluted contrast agent was injected into the tail vein. Repeat injections were performed for each set of 10,000 frames. A 32-gauge needle was used to cannulate the anterior chamber of the eye, which was attached to a balanced salt solution fluid reservoir for control of IOP based on the fluid reservoir height, as seen in Figure 1. The fluid reservoir was in line with a pressure transducer (P75, Harvard Apparatus, Holliston, MA, USA) to confirm in-line pressure to the anterior chamber of the eye.

Ultrasound gel was then applied to the eye as a coupling agent. Prior to MB contrast injection, real-time conventional color doppler mode on the Vevo F2 system at a pulse repetition rate of 6 kHz was used to locate the central retinal artery (CRA) and long posterior ciliary arteries (LPCA), which are located inferior and adjacent to the optic nerve in the rat eye. The scan plane was oriented horizontally (i.e., lateral to medial) and adjusted along each axis to optimize the color doppler image. To mitigate acoustic absorption by the relatively large lens of the rat eye, imaging was performed with the probe placed inferior to the limbus with a pitch of the transducer directed superior towards the optic nerve. The transducer was then adjusted horizontally to optimize centration of the region of interest (ROI) within the image plane. The IOP of the eye was elevated in incremental steps of 10, 20, 30, 40, 50, 60 and back to 10 mm Hg, with a 2-min wait time between each pressure level prior to contrast agent injection and imaging. At each IOP level, 10,000 frames of beamformed RF data were collected and saved for offline post-processing. All 2D imaging was completed within 45 min of anesthesia induction.

### 2.4. Microbubble Localization and Tracking Algorithm

**Figure 2:**
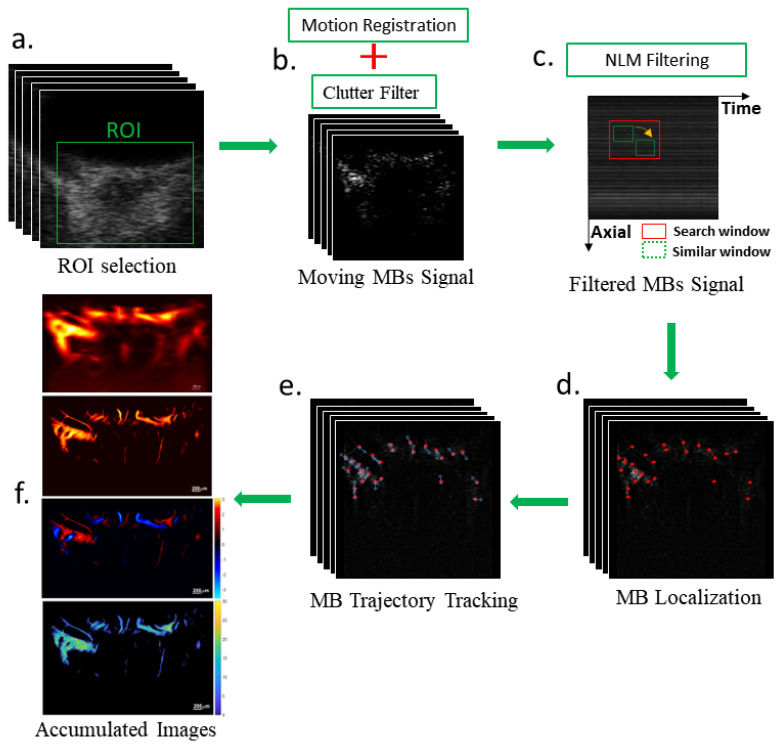
Post-processing steps in the ultrasound localization microscopy algorithm. (**a**) Radiofrequency data was collected for 10,000 frames, (**b**) motion registration and clutter filtering to remove stationary signal, (**c**) spatiotemporal non-local means filter, (**d**) microbubble (MB) localization followed by (**e**) tracking to produce accumulated (**f**) power doppler, density, bi-directional and velocity images.

A schematic of the post-processing steps is shown in Figure 2, with all steps completed in MATLAB 2019a (The MathWorks, Natick, MA, USA). The frames of the RF signal were converted to in-phase quadrature (IQ) data and used to create B-mode images. The ROI was selected to best evaluate perfusion velocity within the LPCAs and CRA.

After selecting the ROI, spatiotemporal singular value decomposition (SVD) was utilized as a clutter filter to separate stationary from moving signals from the ultrasound IQ frames. The IQ data were subdivided into datasets of 1000 frames and reshaped into a 2D Casorati matrix, followed by the SVD calculation. One of the major decision points in SVD filtering is determining the cutoff numbers of the singular values to be retained for the filter. Several techniques have been utilized previously to complete this task, including hard-coding removal of the highest singular values (lowest order values), visually interpreting curvature of the ordered singular values and manually selecting a higher and/or lower cutoff based on the curvature of the ordered values [32,33]. We opted to use an adapted approach from Arnal et al. [34], which determines the singular value cutoffs by analyzing the spatial covariance matrix of the envelopes of spatial singular values where the diagonal is set to zero. Previous methods used a normalized cross-correlation between the covariance matrix and a base of squares to define the singular value of the moving signals. We simplified this approach by finding the maximum difference of the moving median of subsequent rows and columns of the covariance matrix. From this calculation, the stationary signals were reconstructed by selecting the low-order singular values with a subsequent inverse SVD calculation.

The stationary signals were used to estimate tissue motion using a subpixel phase-correlation estimation [35]. The 2D rigid transformation matrix from this calculation was used to correct for motion from frame to frame. Following image registration, power doppler images were created by accumulating the SVD-calculated motion signal along the slow-time dimension. The moving MB signal was then extracted using image subtraction of the registered frames. Given our use of high-frequency ultrasound, we found that the SVD clutter filter produced too high of a density of both moving MB and red blood cell (RBC) signal to track accurately. Image subtraction reduced the moving signal density by better maintaining the contrast difference between the MBs and RBCs, allowing for more accurate tracking of the MB signal. A spatiotemporal non-local means filter was then applied to the IQ data using a window of 9 by 9 pixels and a search window of 17 by 17 pixels with a degree of filtering calculated as two times the standard deviation of a manually selected region of background noise [36,37]. The images were then interpolated to a lateral pixel resolution of 18.1 µm and an axial resolution of 8.2 µm using cubic interpolation (Matlab function ‘interp2’), followed by empirical intensity thresholding. To equalize the brightness of the MBs across the interpolated image, a scalar gaussian filter was applied with an x-dimension value of 3 and a y-dimension value of 1 (Matlab function ‘imgaussfilt’). A 2D normalized cross-correlation was performed on each interpolated frame using a manually selected point-spread function (PSF), resulting in a 2D cross-correlation coefficient map. Pixels with a cross-correlation coefficient below 0.6 were removed and the centroids of each remaining MB signal was calculated using a weighted average approach [32,37,38].

Tracking was performed on the localizations using the Kuhn–Munkres algorithm for assignment with a maximum linkage distance of 130 mm/s, no closing gap and persistence of 5. To remove poor or false tracks, an angle constraint was applied to the tracks to remove pairings with an acute change in direction of over 180 degrees [39]. The tracks were linearly interpolated and smoothed (Matlab function ‘smooth’). Track directions were calculated by determining the relative vertical direction (i.e., towards or away from the transducer) and direction was colored as per traditional color doppler with red towards and blue away from the transducer. The MB tracks were accumulated to generate super-resolved microvessel images consisting of track density, direction and velocity. In order to equalize the weight of systolic and diastolic flow velocities (i.e., a greater number of MB tracks are generated during systole compared to diastole), the mean velocity was determined by calculating the mean of the MB velocities within the selected ROI for each individual frame.

To determine the contrast to noise ratio (*CNR*) of the system with time following MB injection, 3 separate datasets of 10,000 frames were sequentially obtained in one eye, and the *CNR* was calculated over the imaging time for each set of 1000 frames using

(1) $$CNR=\frac{|I_{V}-I_{N}|}{\sigma_{N}}$$

where $I_{V}$ and $I_{N}$ are the mean intensities of a selected vessel and noise ROI, respectively, and $\sigma_{N}$ is the standard deviation of the noise ROI. Approximately 17 s elapsed between each batch of 10,000 frames to allow transfer and saving of the RF data.

The mean diameter of one LPCA and 2 retinal arterioles per eye was calculated using 3 cross-section full width at half maximum (FWHM) measurements at 10 mm Hg. A 3D dataset was also collected in a subset of eyes using the built-in linear motor of the Vevo F2 system with a step-size of 90 µm. A collection of 10,000 frames per step was achieved over a 2.0 mm scanning range. Following all experiments, the animals were euthanized and eyes were collected for further histological analysis.

### 2.5. Statistical Analysis

A one-way analysis of variance (ANOVA) with post-hoc Dunnett’s test was used to calculate the statistical significance of percent perfusion velocity changes with IOP elevation within the LPCA with respect to baseline. Similar analyses were performed for the absolute velocity for the LPCA with respect to baseline. Analyses was performed using R version 4.2.2.

## 3. Results

### 3.1. Controlled Flow Results

**Figure 3:**
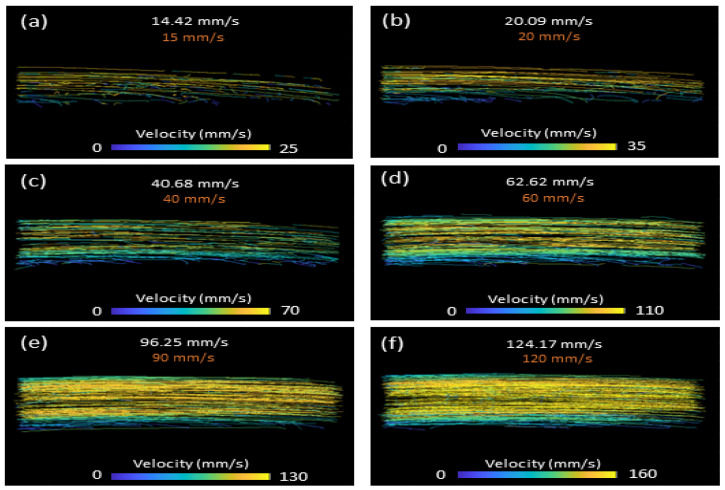
Super-resolution velocity profiles for controlled velocities of (**a**) 15 mm/s, (**b**) 20 mm/s, (**c**) 40 mm/s, (**d**) 60 mm/s, (**e**) 90 mm/s and (**f**) 120 mm/s. The transducer probe was oriented parallel to the flow through the tube.

The reconstructed velocity images of the flow phantom for selected flow speeds are shown in Figure 3. The mean calculated velocity for each selected control velocity of 10, 15, 20, 40, 60, 90, 120 and 150 mm/s were 9.3, 14.4, 20.1, 40.7, 62.6, 96.2, 124.1 and 157.7 mm/s, respectively. All measured velocities fell within 8% of the pre-defined velocities. Laminar velocity profiles were also observed, becoming more evident as velocity was increased. Given that the MB concentration and imaging time remained constant during controlled velocity changes, the density of the MB tracks also increased as velocity increased.

### 3.2. In Vivo Rat Eye Vasculature Imaging

**Figure 4:**
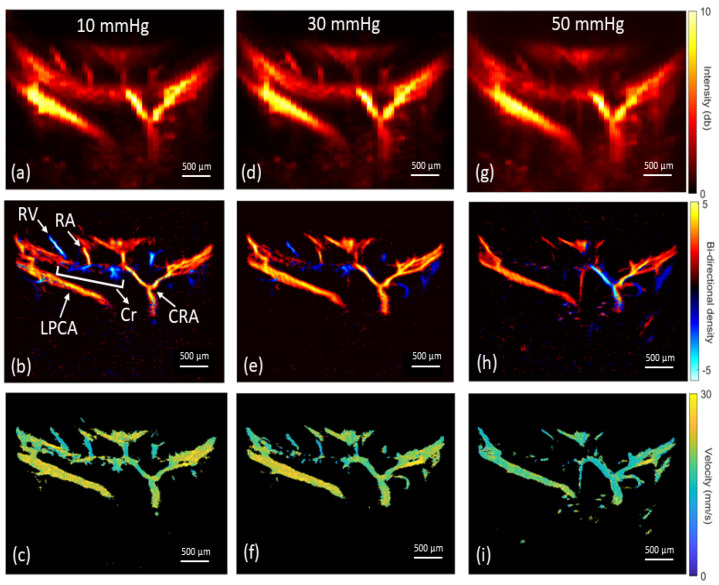
Representative reconstruction of in vivo rat eye vasculature from measurements under increasing IOP magnitudes. Row 1: power doppler images; row 2: bi-directional microvascular images; row 3: flow velocity images. Corresponding images for IOP magnitudes of (**a**–**c**) 10 mm Hg, (**d**–**f**) 30 mm Hg and (**g**–**i**) 50 mm Hg. (**b**) The central retinal artery (CRA) and its branches, long posterior ciliary artery (LPCA), choroid (Cr) and retinal arterioles (RA) and venules (RV) are labeled with arrows.

**Figure 5:**
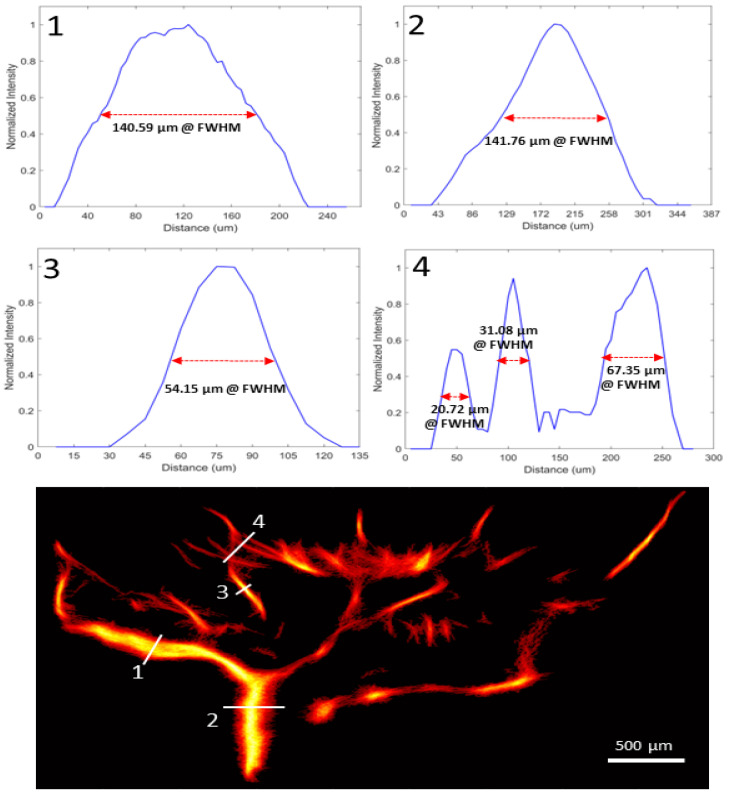
Vasclular density image of representative eye at 10 mm Hg (**bottom**). The 1D cross-sectional intensity profile of the four line markers (**top**) with corresponding full width at half maximum measurements (FWHM).

**Figure 6:**
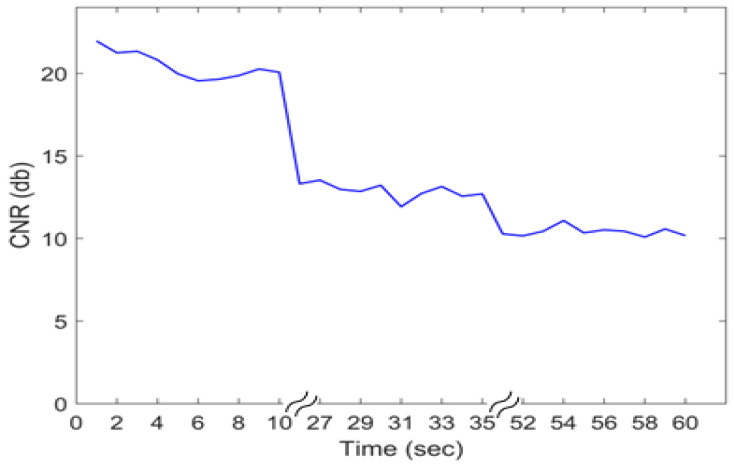
The contrast to noise ratio (CNR) calculate for each set of 1000 frames following microbubble injection. A significant drop in the CNR was noted after the first 10,000 frames (10 s).

The ROI (i.e., LPCAs and CRA) ultrasound depth ranged from 7 mm to 10 mm, with further post-processing cropping to a total vertical imaging height of approximately 3 mm. Representative reconstructed vasculature images are shown in Figure 4, including power doppler, bi-directional and velocity vascular images. Specific vessels can be located from these images based on the known angioarchitecture of the rat eye (Figure 4b), including the LPCA and its branches, CRA, retinal arterioles, choroid, retinal venules and ophthalmic artery. Venules were clearly differentiated from arterioles based on flow direction and velocity. The SRULM images produced vessel measurements with full width at half maximum (FWHM) as low as 20 µm, as seen in Figure 5. The mean diameters of the LPCA and retinal arterioles were 139.8 ± 20.6 µm and 50.0 ± 6.4 µm, respectively. The mean arterial velocities at an IOP of 10 mm Hg within the ophthalmic artery, LPCA, LPCA primary branches, CRA and retinal arterioles were 22.5 ± 4.2, 30.1 ± 4.3, 21.7 ± 3.8, 21.5 ± 3.8 and 14.7 ± 3.3 mm/s, respectively. The mean velocity within the retinal venules was 10.7 ± 3.1 mm/s and CRV was 13.7 ± 4.6 mm/s at an IOP of 10 mm Hg. A reduction in CNR, as seen in Figure 6, was noted over time as MBs were rapidly cleared from the vasculature.

### 3.3. Vascular Perfusion Changes with Elevated IOP

**Figure 7:**
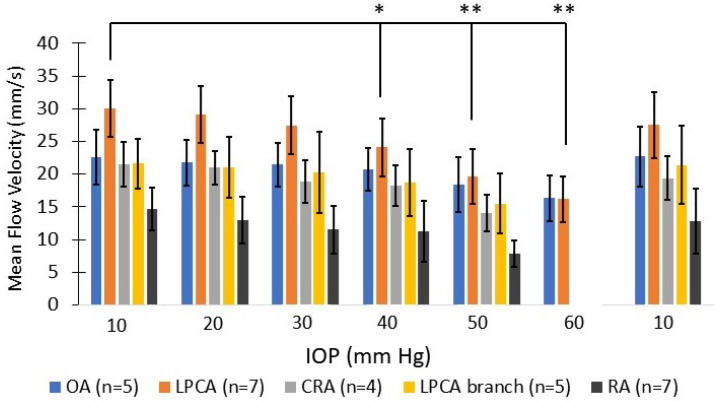
Mean absolute arterial perfusion velocity (error bars represent standard deviation) for selected vessels at each IOP level. OA = ophthalmic artery; LPCA = long posterior ciliary artery; CRA = central retinal artery; RA = retinal arteriole. * indicates *p* ≤ 0.05; ** indicates *p* ≤ 0.001. Statistical analysis completed for LPCAs.

**Figure 8:**
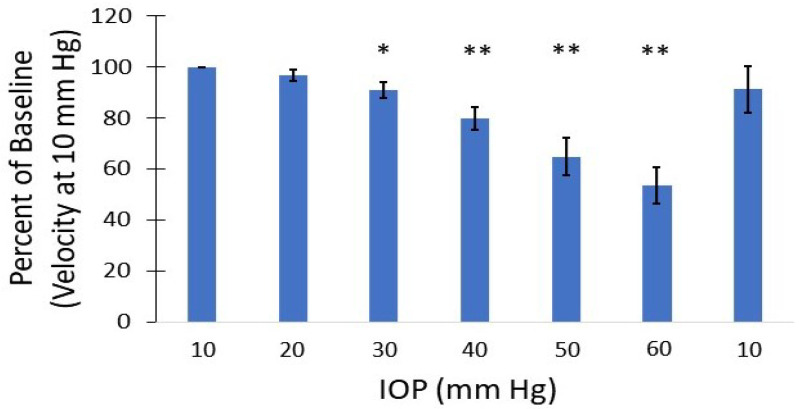
Mean percentile reduction in velocity of the long posterior ciliary artery with respect to baseline velocity at 10 mm Hg. * indicates *p* ≤ 0.05; ** indicates *p* ≤ 0.001.

The mean velocity of selected vessels as IOP was incrementally increased are shown in Figure 7. Vascular density also reduced at IOP levels greater than 30 mm Hg. At IOP magnitudes of 50–60 mm Hg, a reversal of the mean blood flow direction was noted in some arteries, as seen within the CRA in the bi-directional flow map at 50 mm Hg (Figure 4h). Given the combination of flow reversal and reduced MB signal, particularly in the smaller arteries, the mean velocity at 60 mm Hg could only be calculated for the ophthalmic artery and LPCA. The greatest velocity reductions occurred within the retinal arterioles as IOP was elevated, with a mean reduction of 46.7% in velocity at an IOP of 50 mm Hg compared to 10 mm Hg. Although several retinal arterioles and venules were no longer visible at IOP magnitudes of 50 to 60 mm Hg, major arteries (e.g., CRA, LPCA) remained visible at the maximum IOP of 60 mm Hg. The percent reduction in perfusion velocity from the baseline IOP of 10 mm Hg for the LPCA is shown in Figure 8. A statistically significant percent reduction in the velocity occurred in the LPCA, beginning at 30 mm Hg, with a 47.5 ± 7.1% reduction from baseline at the maximum IOP magnitude of 60 mm Hg. All perfusion velocities returned to baseline when IOP was lowered back to 10 mm Hg. A reduction in CNR was also noted at elevated IOP, particularly during diastole (see Supplemental Video S2 taken at 50 mm Hg).

### 3.4. 3D Volume Imaging

**Figure 9:**
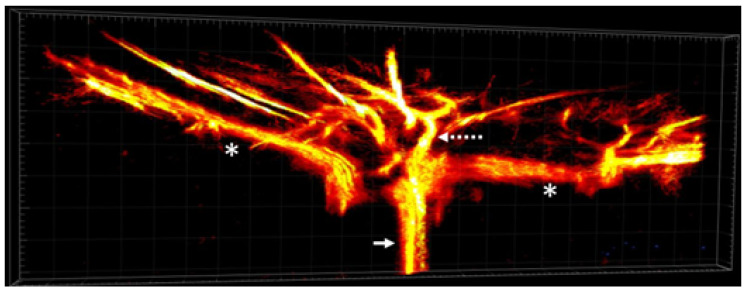
The 3D volume reconstruction of the major rat eye arteries, including the long posterior ciliary arteries (*), ophthalmic artery (arrow), and the central retinal artery (dashed arrow).

Using the vascular density images from each 2D scan, 3D data were reconstructed in Imaris Viewer (Oxford Instruments, Abingdon, UK) as seen in Figure 9. The anatomy of the major vessels of the posterior eye are better visualized with the 3D reconstruction, demonstrating the ophthalmic artery, its trifurcation into the nasal and temporal LPCAs and the CRA. Further branches of these vessels can also be visualized.

## 4. Discussion

Using SRULM, we were able to discriminate the morphology and flow velocity of several vessels of interest within and behind the posterior globe of the rat eye. The in vivo anatomical features of the vasculature from our study corroborated with prior ex vivo vascular casting and histological studies of the rat posterior globe angioarchitecture [40,41,42]. Immediately behind the posterior sclera and inferior to ONH insertion, the trifurcation of the ophthalmic artery into two LPCAs and CRA was evident during imaging and was used as an anatomical landmark. Using three measurements from each eye, the mean diameter of the LPCA in our study was 139.8 ± 20.6 µm. Bhutto et al. [41], using electron microscopy of vascular casts of the rat eye, measured the LPCA diameters between 110 and 126 µm, with these vessels giving off 5–7 branches at a right angle to the choroid. The diameters of the retinal arteries (50.0 ± 6.4 µm), originating from the ONH in a spoke wheel pattern, also appeared to be consistent with prior optical measurements that ranged from 30–60 µm [43,44,45]. It is important to note that, in the rat, branches of the LPCA supply blood flow to the majority of the optic nerve transition zones, while in humans several short posterior ciliary arteries branch off the ophthalmic artery to penetrate the sclera and form the circle of Zinn–Haller to supply this region of the ONH.

The mean arterial velocities at 10 mm Hg in this study were comparable to prior studies in the rat using doppler ultrasound, SRULM and optical erythrocyte tracking techniques. Using arterial spectrograms, Silverman et al. [24] found a mean arterial velocity of 30.9 ± 10.8 mm/s at 10 mm Hg within the LPCA in the rat eye. Morisset et al. [33], utilizing SRULM, measured flow velocities of 25 mm/s in the ophthalmic artery, 30–40 mm/s in the LPCAs and 20 mm/s in the CRA. First-order arterioles in their study had velocities up to 23 mm/s. Tracking fluorescently labeled erythrocytes to measure in vivo flow velocity in the rat eye, Kornfield et al. [43] measured velocities in the range of 14–20 mm/s in retinal arterioles and 8–12 mm/s in venules, depending on the location of the erythrocyte across the diameter of the vessel.

Several studies have shown statistically significant perfusion reductions to the retina and retrobulbar vasculature in the rat eye at IOP magnitudes starting around 30–50 mm Hg [24,45,46]. We also found that our SRULM method was sensitive to perfusion velocity changes of the posterior globe of the rat during IOP elevation. Mean arterial velocities within the LPCA were reduced by 47.5 ± 7.1% at an IOP of 60 mm Hg. Silverman’s group showed a reduction of mean arterial velocity of approximately 65% in the LPCA for the same IOP elevation in Sprague–Dawley rats [24]. The smaller reduction in our study may be secondary to methodology or the location in which the velocity measurements of the LPCA were taken. The LPCA gradually penetrates the sclera as it travels along the globe [42]. The portion of the LPCA our measurements were derived from were posterior and external to the sclera. It is possible that measurements taken from the intraocular LPCA could demonstrate greater velocity reductions secondary to direct compression as IOP is elevated. The mean velocity of the retinal arterioles in our study reduced to 53.3% of baseline at an IOP of 50 mm Hg, which was similar to flow reductions of the same vessels in rats using optical measurements in these vessels [45]. We also found that perfusion velocity returned to normal when IOP was reduced back to 10 mm Hg from 60 mm Hg.

**Figure 10:**
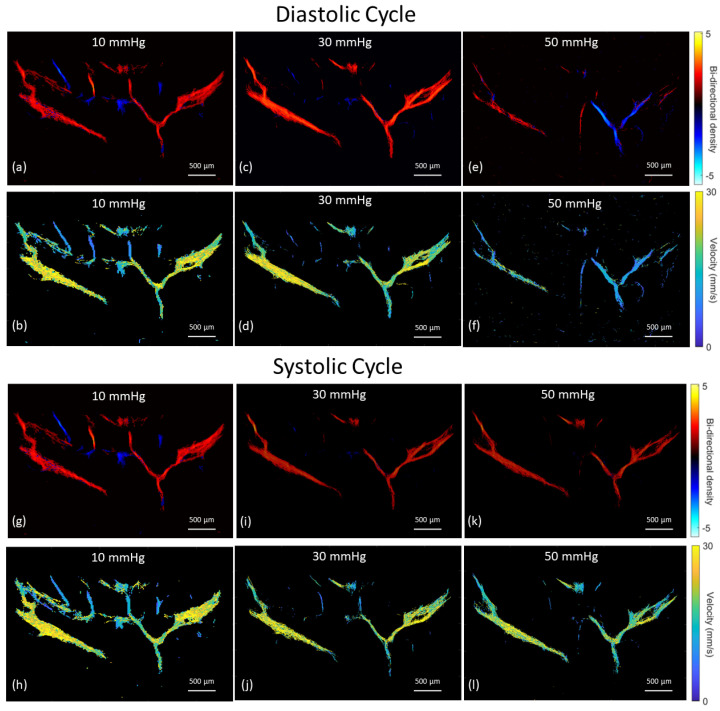
Bi-directional flow and velocity images reconstructed using microbubble tracks extracted from the systolic and diastolic frames defined by cutoff ranges of the synchronized electrocardiogram readings. Diastolic images are shown in the top two rows for IOP magnitudes of (**a**,**b**) 10 mm Hg, (**c**,**d**) 30 mm Hg and (**e**,**f**) 50 mm Hg. Systolic images are shown in the bottom two rows for IOP magnitudes of (**g**,**h**) 10 mm Hg, (**i**,**j**) 30 mm Hg and (**k**,**l**) 50 mm Hg.

One of the more interesting and unexpected findings from this study was a notable reversal of blood flow in major arteries such as the CRA at IOP magnitudes of 50–60 mm Hg. This was not overly apparent in the analysis of the mean bi-directional track images but was clear when evaluating the post-processed videos of MB flow (Supplementary Video S2 at 50 mm Hg compared to Supplementary Video S1 at 10 mm Hg). For this reason, we attempted to use the synchronized ECG data available for one eye to extract systolic and diastolic frames to better assess this reversal of flow that appeared during diastole. Frames within the time period beginning at the peak of the T-wave and ending with the peak of the P were classified as systolic frames. Tracking was then performed separately on the systolic and diastolic frames. As seen in Figure 10, a clear reversal of flow is present during diastole, but not systole, within the CRA at an IOP of 50 mm Hg. Given that the mean diastolic aortic blood pressure in rats anesthetized with 2–3% isoflurane is between 49.4 ± 23.1 (3%) and 62.5 ± 30.1 mm Hg, as IOP elevated above 50 mm Hg, the diastolic perfusion pressure to the eye approaches zero [47]. This resulted in a reversal of blood flow direction in certain arteries during diastole, as seen in Figure 10. Blood pressure was not measured during our experiments and is planned for future studies. Qualitatively, it appeared that the major arteries with a more perpendicular penetration approach to the sclera (e.g., CRA) underwent flow reversal more often compared to the LPCAs, which enter the sclera gradually at an oblique angle [42]. We also found a reduction in the CNR of the MBs at IOP magnitudes of 50–60 mm Hg. This was particularly evident during diastole (Supplementary Video S2), when the MB flow slowed and reversed direction. The reduced contrast may result from a combination of reduced volume of MBs and constraints in MB expansion as vessels are compressed.

Reversal of blood flow with IOP elevation has been seen in human retinal arteries with central retinal vein occlusions and elevated IOP using laser doppler holography [48]. Consistent with our study, using visible light optical coherence tomography, reversal of retinal arterial blood flow was noted in 4-month-old Brown Norway rats at IOP magnitudes of 50 to 60 mm Hg [46]. Interestingly, retinal function in rats measured via electroretinograms (ERGs) appears to remain normal during acute IOP elevations up to 50 to 60 mm Hg, despite reductions in blood flow [49]. This phenomenon is likely related to increased oxygen exchange at lower flow rates, as the difference between oxygenation of the arterial and venous retinal vessels increases exponentially, beginning at IOP magnitudes of 30–40 mm Hg [46]. However, the reversal of arterial blood flow occurring around 50 mm Hg may indicate a tipping point for inducing tissue hypoxia, resulting in the ERG changes seen at these IOP levels. Future studies incorporating measurements of tissue hypoxia or ischemic damage may provide insight into this phenomena.

One of the major limitations of our SRULM technique is its inability to measure volumetric flow rates. We have demonstrated the ability to obtain 3D volumes; however, this is simply a volumetric reconstruction of 2D data. More recently, 2D array transducers have been utilized for microvasculature mapping of the brain in 3D, allowing the ability to track MBs in all dimensions [50]. The 2D limitation of this technique also makes evaluating velocity changes with acutely elevated IOP via needle cannulation experimentally difficult, as slight shifts in the location with IOP elevation may reduce the repeatability of the scanning plane. Similarly, repeating measurements at the same ROI during longitudinal studies without the aid of registered 3D volumes would be difficult. For this reason, future experiments will utilize 3D data collection in order to confirm the repeatability of the ROI and investigate volumetric flow. Experimentally, we found that shadowing from the lens of the rat eye sometimes limited imaging the entire vasculature surrounding the ONH. We partially overcame this by imaging at an inferior to superior angle; however, removal of the superior orbital bone may provide the best visualization of the entire ONH region. The influence of anesthetic type has also been shown to influence total retinal blood flow (TRBF) measured using doppler OCT, with 1.5–2.5% isoflurane anesthetized rats having approximately twice the TRBF as rats measured under anesthesia with intraperitoneal ketamine-xylazine [51]. This result was thought to be related to the vasodilatory effect of isoflurane. This vasodilation may alter ocular blood flow and its autoregulation to elevations in IOP, which would make the current findings less physiologically relevant. We plan to examine the influence of various anesthetics on our flow velocity metrics in the future. Injection of the MB contrast agent greatly aided in increasing the CNR during imaging. However, the CNR reduced during image acquisition as the perfluoropropane MBs used in these experiments were cleared relatively quickly from the rat, requiring injection prior to every 10,000 frame collection. Future work will utilize perfluorobutane MBs to reduce the frequency of injections during imaging acquisition.

## 5. Conclusions

We present a novel approach for ocular vascular imaging in rodent eyes, with the ability to quantify vascular perfusion velocities that cannot be measured with current optical techniques. Morphological and functional results were consistent to prior studies that characterized the vasculature of the rat eye. We demonstrated that this technique is capable of measuring the changes in ocular blood flow as IOP is elevated. The relatively short image acquisition time provides the potential for 3D applications that will improve anatomical localization of the angioarchitecture around the rat ONH. The penetration depth compared to optical techniques (e.g., optical coherence tomography and laser doppler flowgraphy) and improved resolution compared to standard doppler ultrasound provides the potential to assess the influence of IOP on ocular blood flow in addition to quantifying potential therapeutics with the goal of increasing perfusion to the eye.

## Data Availability

The data presented in this study are available on request from the corresponding author.

## Supplementary Materials

The following supporting information can be downloaded at: https://www.mdpi.com/article/10.3390/bioengineering10060689/s1, Video S1: Processed microbubble flow video through the posterior rat eye at IOP magnitude of 10 mm Hg; Video S2: Processed microbubble flow video through the posterior rat eye at IOP magnitude of 50 mm Hg.
